# Oxidative Stress in Plasma from Patients with Marfan Syndrome Is Modulated by Deodorized Garlic Preliminary Findings

**DOI:** 10.1155/2022/5492127

**Published:** 2022-01-17

**Authors:** Israel Pérez-Torres, María Elena Soto, Linaloe Manzano-Pech, Eulises Díaz-Diaz, Elizabeth Soria-Castro, María Esther Rubio-Ruíz, Verónica Guarner-Lans

**Affiliations:** ^1^Department of Cardiovascular Biomedicine, Instituto Nacional de Cardiología “Ignacio Chávez”, Juan Badiano 1, Sección XVI, Tlalpan, México City 14080, Mexico; ^2^Department of Immunology, Instituto Nacional de Cardiología “Ignacio Chávez”, Juan Badiano 1, Sección XVI, Tlalpan, México City 14080, Mexico; ^3^Department of Reproductive Biology, Instituto Nacional de Ciencias Médicas y Nutrición Salvador Zubirán, Vasco de Quiroga 15, Sección XVI, Tlalpan, México City 14000, Mexico; ^4^Department of Physiology, Instituto Nacional de Cardiología “Ignacio Chávez”, Juan Badiano 1, Sección XVI, Tlalpan, México City 14080, Mexico

## Abstract

Marfan syndrome (MFS) is a genetic disorder of connective tissue that affects the fibrillin-1 protein (FBN-1). It is associated with the formation of aneurysms, damage to the endothelium and oxidative stress (OS). Allium sativum (garlic) has antioxidant properties; therefore, the goal of this study was to show the antioxidant effect of deodorized garlic (DG) on antioxidant enzymes and OS markers in the plasma of patients with MFS. The activity of antioxidant enzymes such as extracellular superoxide dismutase (EcSOD), peroxidases, glutathione peroxidase (GPx), gluthatione-S-tranferase (GST), and thioredoxin reductase (TrxR) was quantified, and nonenzymatic antioxidant system markers including lipid peroxidation (LPO), carbonylation, nitrates/nitrites, GSH, and vitamin C in plasma were determined in patients with MFS before and after treatment with DG. The results show that DG increased the activity of the EcSOD, peroxidases, GPx, GST, TrxR (*p* ≤ 0.05) and decrease LPO, carbonylation, and nitrates/nitrites (*p* ≤ 0.01). However, glutathione was increased (*p* = 0.01) in plasma from patients with MFS. This suggests that treatment with garlic could lower the OS threshold by increasing the activity of antioxidant enzymes and could help in the prevention and mitigation of adverse OS in patients with MFS.

## 1. Introduction

Marfan syndrome (MFS) is a disorder of genetic origin with an autosomal dominant character that affects the gene that encodes for the fibrillin-1 protein (FBN-1), therefore altering connective tissue. It is associated with deformity and dysfunction of elastic fibers, which results in structural and functional damage to the structure of the aorta causing micro dissection of the middle layer and degeneration [1]. Damage to the aortic tissue in MFS is accompanied by oxidative stress (OS), vascular dysfunction, and loss of the contractile function and the endothelium-dependent relaxation [1].

OS is caused by an imbalance between the production of reactive oxygen species (ROS) and the ability to rapidly detoxify their intermediate reagents or repair the damage caused by them through the employment of biological antioxidant systems [2]. Biological macromolecules such as lipids, carbohydrates, proteins, and nucleic acids are altered by instability in their structures caused by their reaction with ROS [3]. Excess ROS and reactive nitrogen species are implicated in cardiovascular disease (CVD). ROS are produced by several pathways including mitochondria, xanthine oxidase, NADPH oxidase, and inducible nitric oxide synthase (iNOS) [4]. There is an inverse association between the risk of degenerative diseases where there is an increase in OS and the consumption of medicinal plants. Therefore, it is important to seek for strategies to improve the antioxidant capacity in these diseases including MFS. One of these strategies implies the use of garlic which has antioxidant properties. The intake of therapeutic medicinal plants that containing antioxidants can reduce oxidation at the endogenous level, thus diminishing the negative consequences derived from OS [3]. Recent work indicates that cursive sativum (garlic) has antioxidant properties and reduces the OS present in CVD [5]. Deodorized garlic (DG) extracts contain phytochemicals and lipid-soluble organ sulfur compounds, such as dial-lyl-thiosulfonate (allicin) and selenium that protect against OS [6]. DG in tablets has fewer harmful side effects than raw garlic [6]. Furthermore, DG has beneficial effects for treatment of ROS-mediated CVD [7].

DG stabilizes compounds with antioxidant properties such as allicin, S-allyl-cysteine (SAC), and S-allylmercaptocysteine (SAMC) [5]. These stable compounds exert antioxidant actions by eliminating ROS, increasing the activity of cellular antioxidant enzymes such as superoxide dismutase superoxide dismutase (SOD) isoforms, catalase and GPx, and GSH levels. In a review where garlic supplementation was analyzed and that included four meta-analyses, garlic reduced systolic and diastolic blood pressure. In another study using garlic supplementation, there was a reduction of total cholesterol [8]. Furthermore, DG had more consistent benefits than raw garlic, and rare adverse reactions were documented with established limited causality [9].

The participation of OS in the progression of aortic damage in MFS has been described [10]. LPO causes vasomotor dysfunction in the thoracic aorta associated with OS, which is related to a decrease in eNOS and an increase in the iNOS pathways, and a decrease in the activity of the superoxide dismutase (SOD) isoforms [11].

Due to the aforementioned information and to the lack of reports on the antioxidant properties of DG on OS in patients with MS, the goal of this study was to show the antioxidant effect of DG in the plasma of MFS patients.

## 2. Materials and Methods

### 2.1. Population in Study

This was a controlled, open, analytical, prospective, and longitudinal (before-after) study that included 13 patients of either sex, 6 men, and 7 women, that were admitted to the Ignacio Chávez National Cardiology Institute consecutively with aortic root dilation (>50 mm). The dilation was demonstrated by magnetic resonance angiography.

The cases included MFS patients, classified by the Ghent criteria in 1996 [12]. Once the patients completed the inclusion criteria, a cardiological investigation of the clinical condition of each patient was analyzed, including various clinical indications of their cardiac pathology.

Studies including clotting times, radiographs, electrocardiography, anesthetic evaluation, and current medical treatment were obtained, and care was taken that the cases were not under treatment with antioxidants, allopurinol, or inhibitors of the probable pathways involved in the production of ROS. Exclusion criteria taken into account were a doubtful diagnosis and/or the lack of agreement to sign the informed consent form for the research study.

Each patient was explained and asked for their approval to include their plasma in this project, doubts were clarified, and an informed consent was obtained to take a blood sample (baseline) and another subsequent blood sample two months after starting the DG treatment when the intervention was concluded.

### 2.2. Healthy Subjects

The control group consisted of 13 healthy men and/or women, who were previously evaluated by an expert cardiologist and rheumatologist to verify that they did not have MFS. Routine laboratory tests were performed to determine triglycerides and cholesterol-HDL, cholesterol-LDL, glucose serum creatinine, and total cholesterrol. In addition, echocardiography, computed tomography, or magnetic resonance imaging were performed to rule out aortic damage. Healthy subjects (HS) were not taking anti-inflammatory drugs, antioxidants, DG, or statins. Medications that could interfere with the outcome of the study, such as non steroidal antiinflammatory drugs NSAIDs, lipid-lowering drugs, and antioxidant supplements were discontinued.

### 2.3. Ethical Considerations

The research protocol was approved by the Research and Ethics Committee of our institution (institutional protocol number: PT-18-101). The study was carried out according to the international ethical standards and the General Health Law, as well as according to the Helsinki declaration, modified at the Congress of Tokyo, Japan, and with informed consent of patients and controls [13].

### 2.4. Treatment

Cursive sativum Chinese garlic tablets (Ajolín Forte® plus, Deodorized Garlic) of 500 mg were ingested orally with water, every 12 hours for 2 months in MFS patients. The nutrition facts of the tablets showed a total fat of 750 mg, 600 *μ*g of sodium, 20 g carbohydrates, and 0 g protein.

### 2.5. Blood Sample Collection

Five ml of blood per patient was collected and centrifuged for 20 min at 936 g at 4°C. The red blood cell pellet was discarded, and the plasma was collected in aliquots of 400 *μ*l and stored at −30°C until used. Blood samples were obtained from each patient before the treatment and after the two months of treatment with the DG.

### 2.6. EcSOD and Peroxidase Activities

The extracellular activity of super oxide dismutase (EcSOD) was determined in plasma by nondenaturing gel electrophoresis and nitro blue tetrazolium (NBT) staining as described by Pérez-Torres et al. [14] 25 *μ*l of plasma was applied directly, without boiling, to a nondenaturing 10% polyacrylamide gel. The electrophoresis was carried out at 120 volts for 4 hours. Subsequently, the gel was incubated in a 2.45 mM NBT solution for 20 min, then the liquid was discarded, and the gel was incubated in a 28 mM EDTA solution, containing 36 mM potassium phosphate (pH 7.8) and 0.028 mM riboflavin. After 10 min of incubation under dark conditions, the nitro blue tetrazolium stain for O_2_^–^ was viewed by UV light exposure for another 10 min. Purified SOD from bovine erythrocytes with a specific activity of 112 U/mg of protein (Sigma-Aldrich, St. Louis, MO, USA) was used as a positive control.

For the activities of peroxidases, 35 *μ*l of horseradish peroxidase was loaded to a final concentration of 178.5 *μ*g as a standard, and 25 *μ*l of plasma in the same conditions of the native gel was run as previously described. To observe the activity of the peroxidases, the gel was washed with distilled water three times, during 5 min, and it was then incubated with a mixture of 3 mg/ml 3,3,5,5-tetramethylbenzidine dissolved in a solution of ethanol: acetic acid: water (1 : 1 : 1) with H_2_O_2_ for 10 min in the dark [15]. In these conditions, where peroxidases are present, the gel remains transparent and 3, 3, 5, 5-tetramethylbenzidine is oxidized showing a green coloration. The gels for EcSOD and peroxidase activities were analyzed by densitometry with a Kodak Image® 3.5 system.

### 2.7. Glutathione Peroxidase and Glutathione-S-Transferase

Glutathione peroxidase and glutathione-S-transferase activities were determinate spectrophotometrically in plasma of each patient as previously described [15]. 100 *μ*l of plasma was suspended in 1.6 ml of 50 mM phosphate buffer (pH 7.3), with added 0.2 mM reduced nicotinamide adenine nucleotide phosphate NADPH, 1 mM GSH, and 1 UI/ml glutathione reductase. The mixture was incubated for 1 min at 37°C, then 100 *μ*l of 0.25 mM H_2_O_2_ was added to start the reaction, and the absorbance was monitored for 6 min at 340 nm. Activity is expressed in nmol of NADPH oxidized/min/ml plasma with an extinction coefficient of 6220 M^−1^ cm^−1^ at 340 nm for NADPH.

For glutathione-S-transferase (GST) activity, 700 *μ*l of phosphate buffer (0.1 M, pH 6.5) supplemented with 100 *μ*l GSH 0.1 mM and 100 *μ*l 1-chloro-2,4-dinitrobenzene (CDNB) 0.1 mM was added to 100 *μ*l of plasma. The sample was incubated and monitored for 10 min at 37°C at 340 nm [16]. Values of GST activity were expressed in U/min/ml of plasma. The GST activity is expressed as units of GS-DNB *μ*mol/min/ml of plasma with an extinction coefficient of 14150 M^−1^ cm^−1^.

### 2.8. Thioredoxin Reductase

Thioredoxin reductase activity (TrxR) was assessed as described previously [17]. 100 *μ*l of plasma suspended in 3 ml of 0.1 mM phosphate buffer (KH_2_PO_4_, pH 7.0) was added to 0.2 mM NADPH, 1 mM EDTA, and 0.1 mg/ml bovine serum albumin. The sample were read in the presence of 20 *μ*l of the specific TrxR inhibitor (10 *μ*M auranofin), and together with a duplicate of the sample without the inhibitor was determined indirectly by the amount of DTNB in the presence of NADPH to form 2 moles of TNB. The DTNB oxidation is monitored at 412 nm at 37°C for 6 min with an extinction coefficient of 13600 M^−1^ cm^−1^.

### 2.9. Lipid Peroxidation

Fatty acids are converted to malondialdehyde which in the presence of thiobarbituric acid develop a pink color which was read at 532 nm. 50 *μ*l CH_3_-OH with 4% BHT plus phosphate buffer pH 7.4 was added to 100 *μ*l of plasma. The mixture was shaken vigorously in vortex for 5 seconds and then incubated in water bath at 37°C for 30 min. 1.5 ml of 0.8 M thiobarbituric acid was then added, and the sample was incubated in a water bath at boiling temperature for 1 hour. After this time and to stop the reaction, the samples were placed on ice; 1 ml 5% KCl was added to each sample as well as 4 ml n-butanol; they were shaken in vortex for 30 sec and centrifuged at 4000 rpm at room temperature for 2 min. Then, the n-butanol phase was extracted, and the absorbance was measured. The calibration curve was obtained using tetraethoxypropane as standard [15].

### 2.10. Carbonylation

Protein carbonylation was detected spectrophotometrically as previously described [15]. 100 *μ*l of plasma were added to 500 *μ*l of HCl 2.5 N. Another sample with 500 *μ*l of 2,4-dinitrophenylhydrazine and incubated in the dark at room temperature for one hour, shaking with a vortex every 15 min, was run in parallel. At the end of the incubation period, 500 *μ*l of 20% trichloroacetic acid were added, and the sample was centrifuged at 15,000 × *g* for 5 min. The supernatant was discarded. Two washings were performed, first removing the precipitate with a sealed capillary tube by adding 1 ml ethanol/ethyl acetate. It was incubated for 10 min, and centrifuging at 15,000× *g* for 10 min. Finally, 1 ml of 6 M guanidine hydrochloride in 20 mM KH_2_PO_4_ pH 2.3 was added. The mixture was incubated again at 37°C for 30 min. Absorbance was read in a spectrophotometer at 370 nm, using water bidistilled as blank and a molar absorption coefficient of 22,000 M^−1^ cm^−1^.

### 2.11. NO_3_^−^/NO_2_^−^ Ratio

The NO_3_^−^ was reduced to NO_2_^−^ by the nitrate reductase enzyme reaction and detected by the technique of the Griess as previously described [15]. The NO_3_^−^ was reduced to NO_2_^−^ by the nitrate reductase enzyme reaction. 100 *μ*l of plasma previously deproteinized with 0.5 N, NaOH and 10%, ZnSO_4_ was mixed, and the supernatant was incubated for 30 min at 37°C in presence of nitrate reductase (5 units). At the end of the incubation period, 200 *μ*l of sulfanilamide 1% and 200 *μ*l of N-naphthyl-ethyldiamine 0.1% were added, and the total volume was adjusted to 1 ml. The absorbance was measured at 540 nm.

### 2.12. GSH Concentration

100 *μ*l of plasma previously deproteinized with 20% trichloroacetic acid (vol/vol) and centrifugated to 10,000 x g for 5 min was added to 800 *μ*l of phosphate buffer 50 mM, pH 7.3, and plus 100 *μ*l of 1 M. The GSH concentration was determined as described previously [15] using Ellman's reagent (5,5′-dithiobis-2-nitrobenzoic acid). The mixture was incubated at room temperature for 5 min, and absorbance was read at 412 nm.

### 2.13. Vitamin C

For measuring vitamin C levels, 20% trichloroacetic acid was added to 100 *μ*l of plasma. After vigorous shaking, the samples were kept in an ice bath for 5 min and centrifuged at 5000 rpm for 5 min; 200 *μ*l of Folin-Ciocalteu reagent 0.20 mM was added to the supernatant. The mixture was shaken vigorously in a vortex for 5 seconds and incubated for 10 min. The absorbance was measured at 760 nm. The calibration curve was obtained using ascorbic acid standard solution [15].

### 2.14. Statistical Analysis

The data are presented as the mean ± SE. Differences were considered as statistically significant when *p* ≤ 0.05. Statistical significance between MFS patients was determined by the Mann–Whitney rank sum test followed by the normality test (Shapiro-Wilk). Statistical significance was determined by one-way ANOVA test, followed by Tukey's post hoc test using Sigma Plot 14 program (Systat Software Inc. 2107, San Jose, CA95131 EE.UU. North First Street, Suite 360).

## 3. Results

### 3.1. General Characteristics

A total of 13 patients with MFS and 13 healthy subjects (HS) were studied. Age in patients with MFS had a median of 26 years with a minimum of 14 and a maximum of 51. The body mass index in MFS patients had a median value of 24 with a minimum of 12 and a maximum of 30. Demographic characteristics of the MFS patients are shown in Table 1, and the blood chemistry of healthy subjects is shown in Table 2. The distribution and frequency of the Ghent criteria of each of the patients are shown in Table 3.

### 3.2. Extra Cellular Superoxide Dismutase and Peroxidase Activities

**Figure 1:**
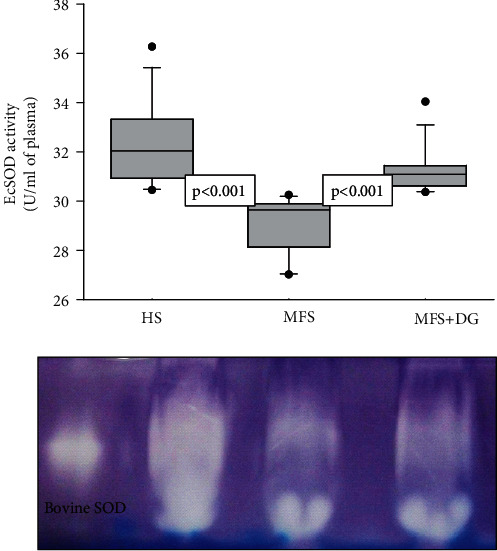
Deodorized garlic increases EcSOD activity in Marfan syndrome patients. HS: healthy subjects; MFS: Marfan syndrome; MFS + DG: Marfan syndrome plus deodorized garlic. (a) is a native gel representative of the EcSOD activity. Riboflavin and TEMED, in the presence of UV light and oxygen, produce superoxide radicals; NBT and SOD compete for them, where SOD is present; the gel remains transparent, whereas reduced NBT turns it purple-blue. The whole scanning shown represents the activity of the enzyme.

**Figure 2:**
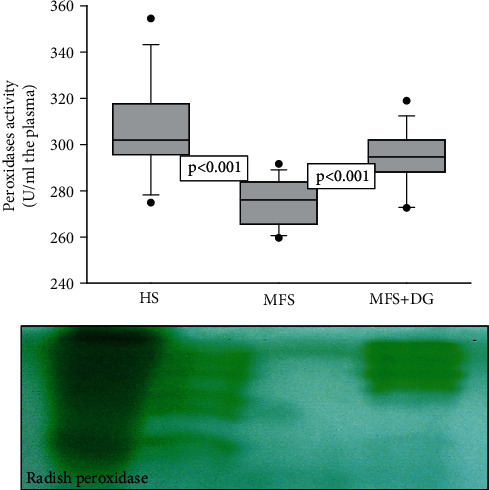
Effect of deodorozid garlic administration on peroxidase activity in healthy subjects and Marfan syndrome patients. A representative native gel is shown below the histogram, where peroxidases are present; the gel remains transparent. and the 3,3,5,5-tetramethylbenzidine is oxidized, showing a green coloration. Abbreviations: HS: healthy subjects; MFS: Marfan syndrome; MFS + DG: Marfan syndrome plus deodorized garlic.

Our results show that EcSOD activity was significantly increased in HS and MFS + DG patients (*p* < 0.001), in comparison to MFS patients (Figure 1). The activity of peroxidases was significantly increased in HS and MFS + DG patients in comparison to MFS patients (*p* < 0.001) (Figure 2).

### 3.3. GPx, GST, and TxrR Activities

**Figure 3:**
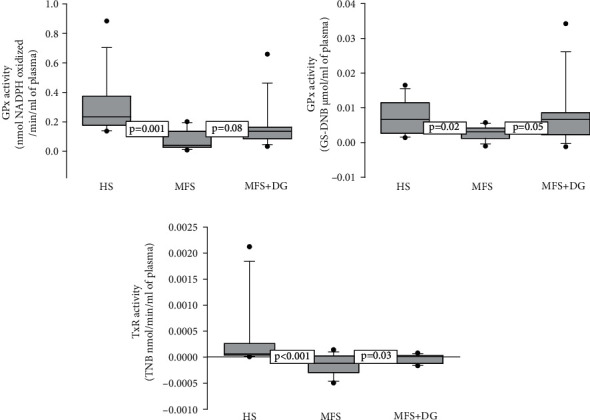
Average activities of GPx (a), GST (b), and TrxR (c) in HS (*n* = 13), MFS patients (*n* = 13), and MFS after DG treatment Abbreviations: HS: healthy subjects; MFS: Marfan syndrome; MFS + DG: Marfan syndrome plus deodorized garlic.

The results of the activity of GPx showed a significant decrease in the MFS patients compared to HS (*p* = 0.001). However, the treatment with DG in MFS patients only showed a tendency to an increase (*p* = 0.08) without reaching a significant value (Figure 3(a)). Our results show that the GST activity was significantly decreased in the MFS patients when compared to HS and MFS + DG patients (*p* = 0.02 and *p* = 0.05, respectively, Figure 3(b)). The TrxR in MFS patients was significantly decreased when compared to that in the HS and MFS + DG subjects (*p* < 0.001 and *p* = 0.03, respectively, Figure 3(c)).

### 3.4. Non Enzymatic Antioxidant System Markers: Lipid Peroxidation and Carbonylation

The OS indicators in the plasma of the experimental groups are shown in Table 3. There was a significant increase in LPO (*p* ≤ 0.001 and *p* = 0.01), carbonylation (*p* ≤ 0.001), and NO_3_^−^/NO_2_^−^ ratio levels in the MFS group in comparison to the HS and MFS + DG groups (*p* ≤ 0.001 and *p* = 0.01, respectively). However, the GSH levels showed a decrease in MFS patients with a significant difference compared to the HS and MFS + DG groups, respectively (*p* ≤ 0.001 and *p* = 0.01). Vitamin C levels showed no significant changes in the groups.

## 4. Discussion

The medicinal use of garlic in folk medicine is extremely old, and thousands of investigations have shown its beneficial effects on different pathologies such as hypertension, dyslipidemias, insulin resistance, and OS to mention a few. The beneficial effects of garlic are due to organ sulfuric compounds, such as allicin, E/Z-ajoene, SAC, S-allyl-cysteine sulfoxide (alliin), SAMC, diallyl sulfide (DAS), diallyl disulfide (DADS), diallyl trisulfide (DATS), gamma-glutamyl tripeptides, and sulfur dioxide(SO_2_) among others [3].

On the other hand, in animal models and in humans with MFS, there is formation of aortic aneurysms and pseudoaneurysms which is accompanied by endothelial dysfunction, chronic inflammation, increase in the expression and activity of metalloproteinase, and a decrease of the antioxidant enzymes. These alterations are due to the genetic mutation of the FBN-1 gene [18].

**Figure 4:**
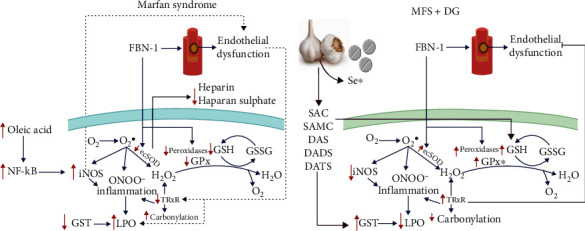
Contributions of deodorized garlic in the antioxidant systems in Marfan syndrome. ^∗^Enzymes stimulated by selenium. Abbreviations: DADS: diallyl disulfide; DAS: diallyl sulfide; DATS: diallyl trisulfide; DG: deodorized garlic; EcSOD: extracellular superoxide dismutase; FBN-1: fibrillin-1 gene; GPx: glutathione peroxidase; GSH: glutathione; GSSG: oxidized glutathione; GST: gluthatione-S-transferase; H_2_O_2_: hydrogen peroxide; iNOS: inducible nitric oxide synthase; LPO: lipoperoxidation; NF-*κ*B: nuclear factor *κ*-light-chain-enhancer of activated B cells; O_2_-: superoxide anion; ONOO−: peroxynitrate; SAC: S-allyl-cysteine; SAMC: S-allylmercaptocysteine; Se: selenium.

The objective of this work was to show the antioxidant effect of DG in the plasma of MFS patient. As far as we know, there are no studies to date that show the beneficial effect of garlic in this syndrome.  Figure 4 summarizes the results of this study on DG treatment in the plasma of the MFS patients.

### 4.1. Superoxide Dismutase

SOD isoforms participate as the first line of detoxification against the O_2_^–^ anion reducing it to H_2_O_2_. This enzyme is expressed in blood vessels primarily on the surface of vascular smooth muscle cells and the subendothelial space. It contains a binding domain that links it to proteoglycans and heparan sulphates which are expressed on the cell surface. This enzyme may be secreted into the extracellular space and is found in plasma [19]. The results in this study suggest that the decrease of EcSOD activity could contribute to OS in MFS patients [20]. The mutation of FBN-1 is also associated with a decrease in heparin/heparan sulphate for which the EcSOD has a binding domain. Genetic factors such as polymorphisms in the heparin binding domain alter the expression or activity of this enzyme [21]. Regarding the alterations of the expression or activity of this enzyme by medicinal plants that provide antioxidants in MFS patients, it was previously found that an infusion of *Hibiscus sabdariffa L.* increased its activity [11]. Our results show that the treatment with DG increases the EcSOD activity in MFS patients. The beneficial effect of garlic is due to the organ sulfuric compounds such SAC and SAMC responsible for the transcription of some antioxidant enzymes such as SOD isoforms through the NrF2 pathway [22]. In addition, this result suggests that the O_2_^–^ anion concentration decreases but that H_2_O_2_ increases.

### 4.2. GPx Activity

To detoxify the H_2_O_2_, the cell antioxidant system is provided with other enzymes that employ it as a substrate. 12% of the EcSOD functions are related to the activity of some of the GPx isoforms [11]. GPx isoforms catalyze the oxidation reaction of glutathione (GSH) to glutathione disulfide using H_2_O_2_. It recycles some of the molecules attacked by H_2_O_2_ and peroxidized organic molecules [23]. However, the activity of GPx is reduced by prooxidative conditions such as an inflammatory state which may induce further the accumulation of ROS [24]. Our results show that the GPx activity in plasma of the MFS patients was decreased as had been previously reported [25], and that the DG treatment tended to increase it and thus contribute to the reduction of OS. Several studies have reported that organ sulfuric compounds from garlic can induce the expression of the GPx gene [26]. Another study showed that garlic administration activated the phosphorylation of the Nuclear factor erythroid 2-related factor 2 which was associated to an increased in the transcription of the GPx genes [27]. The DG treatment can also provide selenium, an essential micronutrient for the catalytic center of the several antioxidant enzymes such as GPx and TrxR, thus favoring an increase in their activity. In patients with the Loeys-Dietz syndrome, a severe variant of the MFS, there is a decrease in GPx, GST, TrxR, selenium, and Nrf2 expression [28]. However, in MFS patients, the involvement of selenium requires further study.

### 4.3. Activity of Peroxidases

Other antioxidant enzymes that contribute to reduce H_2_O_2_ to water are peroxidases. These enzymes play an important role in innate immunity and in other physiologically important processes like apoptosis and cell signaling [29]. Our results show that the activity of peroxidases decreases in MFS patients. This may be due to the FBN-1 mutation since the fibrillins constitute the backbone of microfibrils in the extracellular matrix of elastic and nonelastic tissues [30]. Furthermore, the low activity of these enzymes can contribute to background oxidation which is favored, in part, by the increase in the H_2_O_2_ in MFS patients. Treatment with DG increased the activity of these enzymes, which reduce OS. In HepG2 cells, incubation DAS increases both mRNA and expression of heme oxygenase-1 (HO-1), which is a type deperoxidase [31].

### 4.4. TrxR Activity

TrxR possesses a selenocysteine in its catalytic site [32]. Moderate OS can induce a compensatory increase in the TrxR activity and reduce the oxidative modification of proteins present in several pathologies [33]. However, TrxR is decreased in chronic pathologies with severe OS and metabolic disturbances [34]. In endothelial dysfunction, TxrR decreases, and this is associated with a prothrombotic and proinflammatory state [35]. The thioredoxin system in mammals consists of two antioxidant components, the thioredoxin (Trx) and TrxR. The TrxR enzyme catalyzes the reduction of disulfide in the active site of Trx in the presence of NADPH. It improves the mal function of the proteins, cellular receptors, and/or enzymes [36]. The results of this study suggest that the decrease in TrxR activity in MFS patients could be associated to the background OS. These changes are associated to the lack of disulphide bonds formed among microfibrils where the thioredoxin system is essential to reduce these bridges [37]. However, the DG treatment favored an increase in the activity of this enzyme, which could contribute to decrease the OS and increase the reduction between the disulphide bonds in the microfibrils. The beneficial effect of garlic is associated to organ sulfur compound such the DATS, which modulate the expression and activity of the Trx/TrxR system [37, 38]. These sulfur conjugates may favor an increase in the H_2_S production in the presence of reduced Trx. [38]. H_2_S is a lipophilic molecule that controls important processes in the cell including the regulation of the Keap1-Nrf2 pathway [3].

### 4.5. GST Activity

Another enzyme that showed a decrease in its activity in MFS patients was GST. This is a phase II drug-metabolizing enzyme which detoxifies a wide variety of electrophilic xenobiotics by catalyzing their conjugation to GSH. It also reduces many organic hydroperoxides into alcohols [39]. The decrease in the activity of this enzyme could favor the accumulation of the LPO products including 4-hydroxy-2-transnonenal [40]. However, treatment with DG favored an increase in the activity of this enzyme. Different compounds of garlic are linked to the increase in the activity of this enzyme including DADS and DATS which significantly increased the GST activities in liver damage [41]. Another study demonstrated that organ sulfur compounds increased the activity and mRNA of GST, and this effect was associated to DAS, DADS, and DATS [42]. Furthermore, the reduction of the activities of GST and GPx can also be caused by GSH depletion, since both enzymes depend on it [43].

### 4.6. Redox Biomarkers of the Nonenzymatic System

GSH is the most abundant endogenous intracellular antioxidant present within cells. This tripeptide inactivates the O_2_^−^ anion and the hydroxyl radical. Irreversible cell damage happens when the cell is unable to maintain its intracellular concentration of GSH [44]. Our results show that the GSH concentration was significantly diminished in the MFS patients. However, the treatment with DG increased the GSH concentration favoring the reduction of OS through the provision of a larger amount of this antioxidant molecule that also acts as a substrate for GPx and GST [45]. GSH can be obtained through the diet by consuming foods like garlic, which contains thioallyl compounds such as the DAS DADS and DATS. These compounds maintain the intracellular GSH level modulating its increase and preventing its depletion probably through the enzymes that participate in the GSH synthesis [46].

### 4.7. NO_3_^−^/NO_2_^−^ Ratio, Lipoperoxidation, and Carbonylation

The endothelial dysfunction in MFS patients may inactivate eNOS and increase the iNOS expression/activity leading to an enhanced production of NO which contributes to inflammation [41]. In previous studies in MFS patients, oleic acid was increased, and it may elevate NF-KB which participates in the overproduction of iNOS [47]. Our results show an increase in the NO_3_^−^/NO_2_^−^ ratio in MFS patients. NO metabolites may participate in the chronic inflammation present in this syndrome. Moreover, the loss of the redox homeostasis together with the induction of the iNOS expression/activity and with the subsequent exacerbation of NO through NF-*κ*B activation could lead to the formation of peroxynitrites (ONOO^–^). ONOO^–^ affects protein functions by modifying essential reactive thiol groups and/or tyrosine residues, thereby leading to the formation of oxidized thiol groups or the formation of 3-nitrotyrosine in proteins. These alterations in proteins can induce S-nitrosylation [48]. S-nitrosylation may also regulate the expression of proinflammatory genes. Furthermore, S-nitrosylation impairs both endothelium-dependent and -independent relaxation, and these effects are accompanied by irreversible inactivation of the antioxidant enzymes [49]. However, the treatment with DG favors the inhibition of iNOS [50]. A recent study demonstrated that SAC administration in rats significantly decreased the expression of NF-*κ*B, tumor necrosis factor, and iNOS, exerting a protective effect against toxicity [45]. ONOO^–^ is also an important intermediary in both LPO and carbonylation [51]. We analyzed the LPO and carbonylation levels in our experimental groups. LPO is a marker of damage to cell membranes, and carbonylation is a marker of protein damage by ROS. The DG treatment was able to reduce both indices through its sulfur components by modulating the antioxidant enzymes.

## 5. Conclusions and Perspectives

Our results demonstrated that MFS is associated with the presence of OS, and that the treatment with DG may be effective in diminishing this parameter by increasing the antioxidant defense in the plasma of patients with MFS.

The application of alternative therapies such garlic which have antioxidants properties could help in the prevention and mitigation of adverse OS in the MFS patients and thereby have a beneficial impact on patient survival. These relevant findings suggest the need of conducting multicentric studies or systematic studies providing therapies with antioxidants that may improve the redox state of these patients and that may be appropriate to the clinical context of each particular subject.

## Figures and Tables

**Table 1:** Demographic characteristic Marfan syndrome patients.

	Total	Men (*n* = 6)	Women (*n* = 7)	*p*
Median (min-max)				
Age	26 (14-51)	26 (14-51)	30 (16-42)	
BMI	24 (12-30)	23 (12-25)	24 (18-30)	
(mg/dL)				
Glucose	90 ± 7	92 ± 9	88 ± 5	NS
CrS	0.67 ± 0.17	0.78 ± 0.20	0.58 ± 0.09	NS
CT	166 ± 40	154 ± 24	176 ± 50	NS
HDL	50 ± 13	47 ± 12	53 ± 15	NS
LDL	96 ± 28	93 ± 30	99 ± 29	NS
TG	119 ± 59	122 ± 85	117 ± 32	NS
Score				
Ghent criteria	8 (62)	4 (66.6)	4 (57)	NS
Ectopia lentis	4 (31)	1 (17)	3 (43)	NS
Aortic dilatation	9 (69)	5 (83)	4 (57)	NS
Systemic score	13 (100)	6 (100)	7 (100)	NS
HFA	9 (69)	5 (83)	4 (57)	NS

Abbreviations: BMI: body mass index; CT: cholesterol; CrS: creatinine serum; HDL: high-density lipoprotein; LDL: low-density lipoprotein: HFA: hereditary family antecedent; TG: triglycerides.

**Table 2:** Blood chemistry in healthy subjects.

Glucose (mg/dL)	85.84 ± 5.49
SCr (mg/dL)	0.77 ± 0.02
CT (mg/dL)	174.38 ± 5.56
HDL (mg/dL)	41.23 ± 1.81
LDL (mg/dL)	97.23 ± 4.46
TG (mg/dL)	109.23 ± 15.62

Abbreviations: SCr: serum creatinine; CT: cholesterol; HDL: high-density lipoprotein; LDL: low-density lipoprotein; TG: triglycerides.

**Table 3:** Redox biomarkers of the nonenzymatic system in the plasma.

Parameters (ml of plasma)	HS	MFS basal	MFS + DG
LPO (nmol MDA)	5.25 ± 0.56	11.06 ± 0.69^∗∗^	8.33 ± 0.45^∗^
Carbonylation (nmol carbonyls)	0.08 ± 3.57 × 10^−3^	0.11 ± 3.30 × 10^−3∗∗^	0.09 ± 3.08 × 10^−3∗∗^
NO_3_^–^/NO_2_^–^ (nM)	1.45 ± 0.12	2.46 ± 0.19^∗∗^	1.63 ± 0.19†
GSH (nM)	0.06 ± 2.42 × 10^−3^	0.05 ± 1.76 × 10^−3∗∗^	0.06 ± 4.17 × 10^−3∗^
Vitamin C (*μ*M)	0.20 ± 0.01	0.18 ± 6.70 × 10^−3^	0.19 ± 7.08 × 10^−3^

^∗∗^HS vs. MFS basal, *p* ≤ 0.001, †MFS vs. MFS + garlic, *p* ≤ 0.001, and ^∗^HS and MFS + garlic vs. MFS, *p* = 0.01. Abbreviations: DG: deodorized garlic; HS: healthy subjects; MFS: Marfan syndrome; LPO: lipid peroxidation; NO_3_^–^/NO_2_^–^: nitrate and nitrite ratio; GSH: gluthatione.

## Data Availability

The datasets generated and analyzed during the current study are available from the corresponding author on reasonable request.
